# *Boswellia serrata* dry extract with intestinal anti-inflammatory properties also accelerates gastric ulcer healing in rats

**DOI:** 10.1007/s10787-026-02133-5

**Published:** 2026-04-27

**Authors:** Franco Giovanni Sandri Serafim, Lucas Fontana Breguez da Cunha, Larissa Venzon, Ana Carolina dos Santos Nilz, Bruna Longo, Ruan Kaio Silva Nunes, Max Vidal Gutiérrez, Daniela Miorando, Cristian A. Dalla Vecchia, Walter Antônio Roman Junior, Luisa Mota da Silva

**Affiliations:** 1 School of Health, University of Vale do Itajaí, Itajaí, SC Brazil; 2 Postgraduate Program in Pharmaceutical Sciences, University of Vale do Itajaí, Itajaí, SC Brazil; 3 TGI Pharmacology and Its Interactions Laboratory, Department of Pharmacology, Federal University of Santa Catarina (UFSC), R. João Pio Duarte Silva, 241 - Córrego Grande, Florianópolis, SC 88037-000 Brazil; 4 Department of Chemical, Biological and Agricultural Sciences, University of Sonora, Navojoa Sonora, Mexico; 5 Postgraduate Program in Health Sciences, Community University of Chapecó Region, Chapecó, SC CEP 89809-900 Brazil

**Keywords:** *Boswellia serrata*, Gastric ulcer, Ulcerative colitis, Traditional medicine, Antioxidant activity, Mucosal protection

## Abstract

*Boswellia serrata* is a medicinal plant traditionally used to treat inflammatory conditions and this study aimed to evaluate the efficacy of *B. serrata* dry extract (BSDE) in treating gastric ulcers and ulcerative colitis. Chronic gastric ulcers were induced using 80% acetic acid. For seven days, rats were given oral doses of BSDE (30, 100, or 300 mg/kg), omeprazole (40 mg/kg), or vehicle. Ulcer area, mucin content, and biochemical indicators (GSH, SOD, CAT, GST, MPO, MDA) were evaluated. The rectal administration of 8% acetic acid produced ulcerative colitis. BSDE (100 or 300 mg/kg), dexamethasone (2 mg/kg), or vehicle were given three days before and after colitis induction. Colonic damage was assessed macroscopically and microscopically, as well as mucin production and indicators of oxidative stress. Finally, computational investigations were carried out to identify potential pharmacological targets. BSDE was analyzed using ESI–MS, revealing the presence of six compounds, including α-boswellic acid and keto-boswellic acid. In the gastric ulcer model, oral administration of BSDE for seven days accelerated the ulcer healing, with the 100 mg/kg dose being the most effective. BSDE enhanced gastric mucin content and reduced oxidative stress. In the colitis model, BSDE reduced macroscopic damage, preserved crypt architecture, and decreased inflammatory infiltration. Computational investigations identified potential pharmacological targets for BSDE constituents, including COX-1 and 2, and iNOS. BSDE also demonstrated good ADME features and a favorable toxicity profile. The results suggest that BSDE exerts gastroprotective and intestinal anti-inflammatory effects through antioxidant and mucosal protective mechanisms. These findings support the traditional use of *B. serrata* as a viable therapeutic option for gastrointestinal diseases, highlighting its potential as a phytotherapeutic agent.

## Introduction

Millions of people worldwide suffer from gastrointestinal disorders, which provide serious clinical and financial challenges, which being peptic ulcer disease (PUD) and inflammatory bowel diseases (IBD) the most common of these (Lombardo 2021). Genetic susceptibility, dysbiosis, and dysregulated immune responses to luminal antigens interact intricately in the pathophysiology of IBD (Melo et al. 2021). Although they are not curative, current treatments such as 5-aminosalicylates, corticosteroids, immunosuppressants, and biologics are intended to reduce inflammation and sustain remission (Brasil 2020, GEBID 2019). Long-term adherence is limited by side effects and expensive therapy, underscoring the need for better, more affordable substitutes (GEBID, 2019). Regarding PUD, an imbalance between aggressive factors and mucosal defensive mechanisms leads to this disease (Dong e Kaunitz 2006; Moore 2014) and prolonged use of anti-secretory drugs to treat PUD, such as proton pump inhibitors (PPIs), and H2 receptors antagonists might result in several side effects despite their effectiveness (Araújo 2021).

Prolonged use of anti-inflammatory medicines, particularly non-steroidal ones, is a significant etiological factor of PUD. It is also recognized that steroidal anti-inflammatory drugs can cause delayed gastric healing (Chi 2009). Therefore, those with long-term usage of these drugs due to chronic inflammatory illnesses are more susceptible to gastrointestinal complications (Sochet et al. 2020). Therefore, it is pertinent and would help to innovate the treatment of gastric ulcers to look for an anti-inflammatory agent that does not have ulcerogenic activity or hinder gastric healing.

*Boswellia serrata* Roxb. ex Colebr. (Burseraceae), a tree native to India, Africa, and the Middle East, has been traditionally used in Ayurvedic medicine for centuries to treat chronic inflammatory conditions. Its gum resin, rich in boswellic acids, has demonstrated anti-inflammatory, antioxidant, immunomodulatory, analgesic, and wound-healing activities in nonclinical and clinical studies (Siddiqui 2011; Iram et al. 2017; Ragab et al. 2023). In Western medicine, *B. serrata* is used as an adjunct in osteoarthritis and other chronic inflammatory disorders (Chauhan et al. 2021). Indeed, Boswellic acids, the main bioactive constituents of *B. serrata*, have been shown to interact with 5-lipoxygenase, modulate cytokine production, and scavenge reactive oxygen species (Börner et al. 2023; Safayhi et al. 1995, 1996), making them strong candidates for gastrointestinal inflammation and mucosal healing.

In an acute model of intestinal inflammation, Hartmann et al. (2012, 2014) documented the positive effects of *B. serrata*, showing its capacity to reduce intestinal inflammation. Therefore, given that several anti-inflammatory medications have a side effect of causing stomach injury and that scientific literature points *B. serrata as an* anti-inflammatory natural resource, this work assessed the hypothesis that a commercially available *B. serrata* dry extract that reduces experimental intestinal inflammation is also able to repair gastric ulcerated animals. Our data supported this hypothesis, placing this long-used medicinal species as a viable option in the search for effective anti-inflammatory substances and safe for stomach health.

## Materials and methods

### Study design

The purpose of this quantitative experimental investigation was to assess the anti-inflammatory and anti-ulcer properties of *Boswellia serrata* dry extract (BSDE) in rat models. There were two separate in vivo tests carried out. While the second examined anti-inflammatory effects in a model of acute colitis, the first studied anti-ulcer activity in a model of chronic stomach ulcers. In both models, the induction was made using acetic acid, 80% acetic acid was used to induce stomach ulcers because it is injected into the gastric serosa, causing an inflammatory reaction that leads to the creation of an ulcer in the gastric mucosa. This dose is sufficient for model induction, and similar or greater concentrations have been used in research in this subject, as reviewed by Okabe and Amagase (2005). 8% Acetic acid, on the other hand, is administered intrarectally via a catheter to induce colitis; in this model, the acid comes into close contact with the intestinal mucosa, causing colitis-related damage comparable to that seen by Mohammed et al. (2022). In both models, animals were given BSDE at different doses, a reference medication (positive control), or a vehicle (negative control). Dose selection was based on Hartmann et al. (2012, 2014), who utilized 34.2 mg/kg of a *B. serrata* extract in a model of acetic acid-induced colitis in rats. Therefore, 30, 100, and 300 mg/kg were selected for evaluation in this study. Histological, biochemical and silico analyses were conducted to verify possible modes of action.

### Extract obtaining and dose preparations

The *Boswellia serrata* dry extract, standardized to 65% boswellic acids, was commercially obtained from ACTIVE PHARMACEUTICA LTDA, Brazil (Batch No. 0085/0422). The industrial process typically involves extraction of the resin with a solvent (commonly ethanol), followed by concentration and standardization to achieve the specified 65% boswellic acids content. The commercial supplier provided the standardized extract (65% boswellic acids) used in our study.

The extract was dissolved in the vehicle (water plus 1% tween) to prepare solutions of 30, 100, and 300 mg/mL for administration in rats. Given the average body weights of approximately 250 g for rats, the doses of 30, 100, and 300 mg/kg were administered orally by gavage, adjusting the volume according to the animal’s weight to achieve the desired dose. The administered volume was typically 1 mL/kg, ensuring accurate dosing based on body weight.

### Mass spectrometry analysis (ESI-IT-MS^n^) of BSDE

Before being injected into the mass spectrometry apparatus, the BSDE was dissolved in methanol (MeOH) grade for LC–MS (Liquid Chromatography-Mass Spectrometry) and filtered through a PVDF (Polyvinylidene Fluoride) membrane with a pore size of 0.22 µm. The sample (5 μg/mL) was injected into a Thermo LTQ-XL apparatus coupled to an electrospray ionization source (ESI) and an ion trap analyzer (IT-MS^n^) in negative mode. The analyses were performed at a flow rate of 10 µL/min, with a tube lens voltage of 200 V, a capillary temperature of 280 °C, a spray voltage of 5 kV, a capillary voltage of − 35 V for negative polarity, and a sheath gas flow of 30 arbitrary units. The fragmentations were carried out with helium and a collision energy of 30 eV utilizing the Collision-Induced Dissociation (CID) method.

### Radical scavenging activity test using 2,2-diphenyl-1-picrylhydrazyl (DPPH)

This test evaluates a substance’s antioxidant capacity to sequester the DPPH radical by converting it to hydrazine. It is based on the methodology described by Chen et al. (2004). After diluting the BSDE sample to 1 mg/mL, it was serially diluted at different concentrations (1, 10, 100 and 1000 μg/mL). The methanolic DPPH solution used was 0.3 mM. 750 mL of each sample and 250 mL of DPPH were applied to each well of 96-well microplates to test the antioxidant capacity. Absorbance was measured at 520 nm after 5 min at 25 °C. 50 μg/mL of ascorbic acid served as the positive control, and 750 mL of 0.3 mM DPPH dissolved in methanol and combined with 750 mL of water served as the negative control.

### In vivo experiments

#### Animals

Female Wistar rats (200–300 g), obtained from the Central Animal Facility of the University of Vale do Itajaí (UNIVALI), were used. Animals were housed under controlled conditions (temperature: ~ 23 °C, 12 h light/dark cycle) with ad libitum access to standard chow and water. All procedures followed international guidelines for animal welfare and were approved by the Institutional Animal Care and Use Committee (CEUA/UNIVALI, Protocol No. 024/22).

#### Acetic acid- induced ulcers in rats

**Fig. 1:**
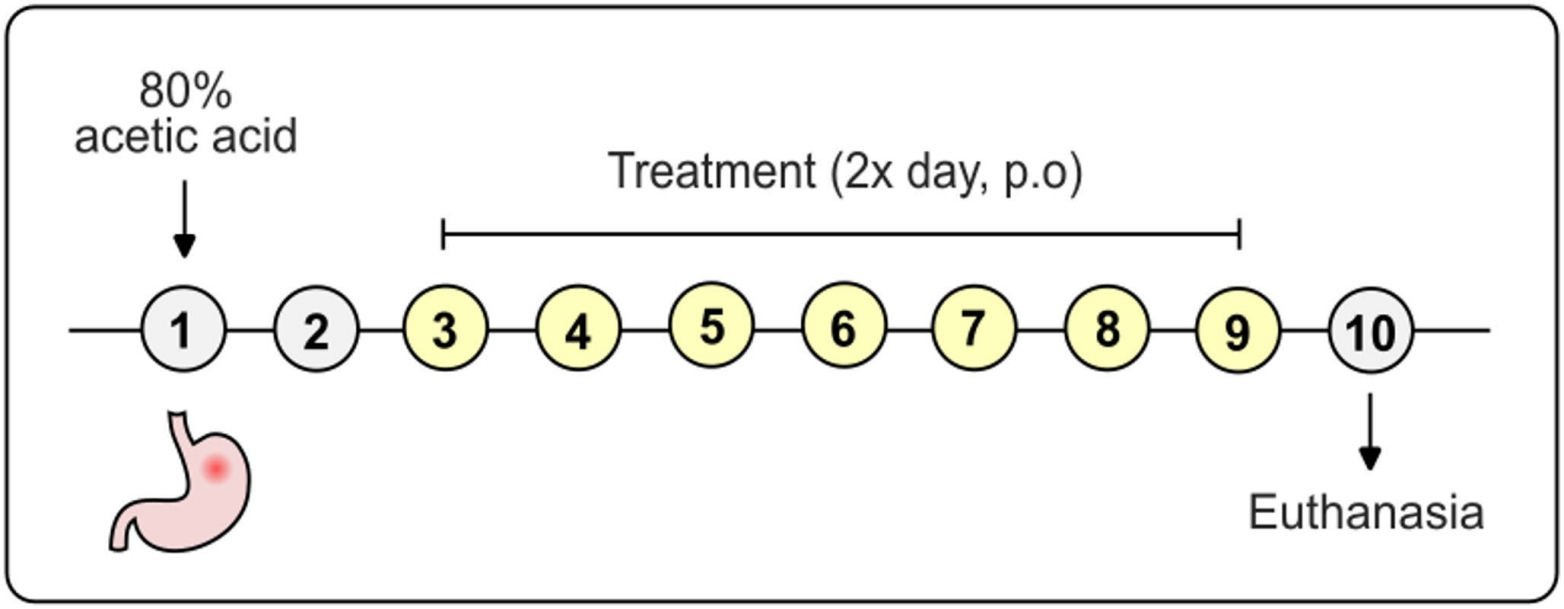
Experimental design and treatment approach for acetic acid-induced chronic gastric ulcer in rats

Gastric ulcers were induced based on the method of Okabe et al. (2005), with minor modifications. Rats fasted for 8 h prior to the procedure were anesthetized (100 mg/kg xylazine plus 50 mg/kg ketamine, i.p.). The abdominal cavity was opened, and a 6 mm glass cylinder was placed on the stomach’s serosal surface. A total of 500 μL of 80% acetic acid was applied for 1 min, followed by rinsing with saline. The abdominal wall was sutured, and animals were maintained under restricted feeding with free access to water. After, the rats were randomly separated into groups (n = 6) and 48 h post- ulcer induction received vehicle (water plus 1% tween, 1 mL/kg), omeprazole (20 mg/kg) or BSDE (30, 100 and 300 mg/kg), orally twice daily for 7 days. At the end of the treatment period, animals were euthanized, and stomachs were excised for macroscopic, histopathological, and biochemical assessments. Ulcer areas in mm^2^ were measured using a graduated ruler. All animals survived after ulcer induction and were included in the study. The experimental design of this experiment was depicted in Fig. 1.

#### Acetic acid- induced colitis in rats

**Fig. 2:**
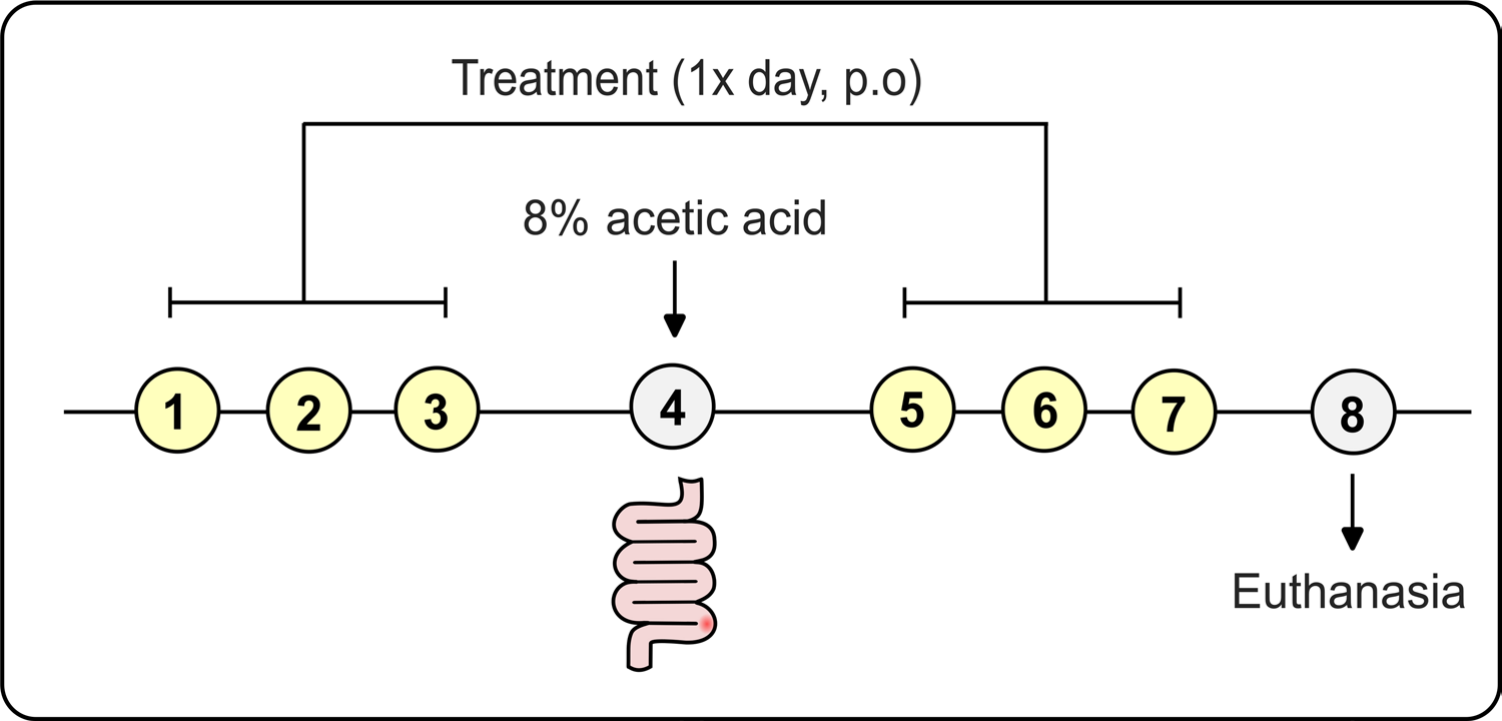
Experimental design and treatment approach for acetic acid-induced colitis in rats

Colitis was induced by intracolonic administration of 8% acetic acid as described by Mann et al. (1980), Yamada et al. (1991) and Mohammed et al. (2022). Rats were fasted for 8 h and anesthetized (xylazine 100 mg/kg plus ketamine 50 mg/kg, i.p.) prior to colonic lavage using 20 mL of 0.9% saline via a polyethylene catheter (6 Fr). After 30 min, 0.5 mL of 8% acetic acid was administered intrarectally at a depth of 6 cm. In this experiment, the treatments were administered once daily for 3 days before and 3 days after colitis induction. Randomly separated groups (n = 6) were administered with vehicle (water plus 1% tween, 1 mL/kg), dexamethasone (2 mg/kg, s.c) or BSDE (100 and 300 mg/kg). Animals were euthanized on day 8, and the colon was harvested for macroscopic, histological, and biochemical evaluation. The experimental design of this experiment was shown in Fig. 2.

### Histological and histochemical evaluation

Following euthanasia, gastric and colonic tissue samples were fixed in a solution composed of 85% ethanol, 10% formaldehyde, and 5% acetic acid for 24 h. Then, the samples were embedded in paraffin and sections of 5 µm thickness were obtained using a microtome. For histopathological assessment, selected sections were stained with hematoxylin and eosin, dehydrated through a graded alcohol series, cleared in xylene, and mounted with coverslips. Microscopic evaluation was performed under light microscopy at 40 × and 100 × magnification. The gastric ulcer samples were evaluated according to Pereira et al. (2013), while the colon slices were evaluated under criteria adapted from Utrilla et al. (2015) and Camuesco et al. (2004), including epithelial integrity, inflammatory cell infiltration, edema, crypt architecture, and goblet cell presence. For mucin detection, sections were processed using the periodic acid-Schiff (PAS) technique, which highlights glycoproteins such as mucins in pink, as performed by Pereira et al (2013) and the PAS-stained regions were quantified using ImageJ software.

### Biochemical analyses

#### Sample preparation

Gastric and colonic tissue samples were collected from the ulcerated or affected sites, weighed, and homogenized in a potassium phosphate buffer (200 mM, pH 6.5). A 50 µL aliquot of the homogenate was reserved for the determination of reduced glutathione (GSH). The remaining homogenate was centrifuged at 9,000 rpm for 20 min at 4 °C. The supernatant was used to assess the activities of superoxide dismutase (SOD), catalase (CAT), glutathione S-transferase (GST) and oxygen species (ROS) levels, while the pellet was reserved for myeloperoxidase (MPO) activity measurement.

#### GSH, ROS and MDA quantification

GSH levels were measured following the method used by Beber et al. (2017). A 50 µL aliquot of homogenate was mixed with 40 µL of 12% trichloroacetic acid in plastic tubes and centrifuged at 4000 rpm for 15 min at 4 °C. For the assay, 10 µL of the supernatant was transferred to a 96-well plate containing 290 µL of TRIS buffer (0.4 M, pH 8.9), followed by the addition of 5 µL of 1 mM 5,5’-dithiobis-2-nitrobenzoic acid. After 5 min, absorbance was read at 415 nm. GSH levels were calculated using a standard curve (3–10 µg/mL) and expressed as µg GSH/g of tissue.

The MDA content, a marker of lipid peroxidation, was measured based on the reaction with thiobarbituric acid (TBARS) as described by Percário et al. (1994). Tissues were mixed with an acidified solution of 75 mM KH_2_PO_4_ (pH 2.5) and then reacted with 10 mM TBA. Samples were incubated at 94 °C for 1 h in a water bath, cooled to 25 °C, and centrifuged at 6000 rpm for 10 min. Absorbance was measured at 535 nm in a 96-well plate. Results were expressed as mmol MDA/g tissue.

#### SOD, CAT and GST activities

SOD activity was determined using the method used by Dieterich et al. (2017), which evaluates inhibition of pyrogallol autoxidation. The reaction mixture consisted of 443 µL Tris–EDTA buffer (0.1 M, pH 8.0) and 20 µL of the sample. After mixing, 25 µL of 1 mM pyrogallol was added, and the solution was incubated for 20 min. Absorbance was measured at 420 nm. One unit (U) of SOD was defined as the amount of protein required to inhibit autoxidation by 50%, and results were expressed as U/mg protein.

CAT activity was assessed according to Cohen et al. (1970). Briefly, 10 µL of tissue homogenate was added to 990 µL of a reaction solution containing 20 mM H_2_O_2_ and 200 mM Tris–EDTA buffer (pH 8.5). The rate of hydrogen peroxide decomposition was measured by absorbance at 240 nm. Results were expressed as mmol H_2_O_2_ decomposed/mg protein.

GST activity was determined using the method described by Habig et al. (1974). A 50 µL aliquot of supernatant was incubated with 205 µL of reaction mixture (1 mM CDNB, 1 mM GSH, and 100 mM potassium phosphate buffer, pH 6.5) in a 96-well plate. Absorbance at 340 nm was recorded for 90 s. Specific activity was calculated using an extinction coefficient of 9.6/mM/cm and expressed as mmol/min/mg protein.

According to Brandt and Keston (1965) and Driver et al. (2000), the fluorescent probe 20,70-dichlorofluorescein-diacetate (DCFH-DA) was used to assess the production of intracellular reactive oxygen species (ROS) in the gastric mucosa. Finally, the fluorescent probe DCFH-DA was used to assess the production of intracellular reactive oxygen species (ROS) in the gastric mucosa, as described by Da Silva et al. (2013). For 40 min, at 37 °C and in the dark, gastric mucosa homogenates from the different groups were incubated with 1 mM DCFH-DA. A luminescence spectrometer with an excitation wavelength of 488 nm and an emission wavelength of 520 nm was used to measure the fluorescence intensity.

#### MPO activity

MPO activity was evaluated according to the method employed by Bradley et al. (1982). The pellet was homogenized in 1 mL of 80 mM potassium phosphate buffer (pH 5.4) containing 0.5% hexadecyltrimethylammonium bromide. After centrifugation at 12.000 rpm for 20 min at 4 °C, 30 µL of the supernatant was added to a 96-well plate along with 220 µL of reaction buffer (containing phosphate buffer and H_2_O_2_and 20 µL of 18.4 mM tetramethylbenzidine. After incubation at 37 °C for 3 min, the reaction was stopped with 30 µL of 1.46 M sodium acetate (pH 3.0). Absorbance was read at 620 nm. Results were expressed as OD units/mg protein/3 min.

Furthermore, the precipitation from a homogenized sample of a vehicle-treated ulcerated rat with high MPO activity was used to assess the direct impact of BSDE on MPO activity in vitro. MPO activity was measured and expressed as previously described after the resulting supernatants were treated with BSDE (1, 10, and 100 μg/mL) for 15 min at 25 °C (Bradley et al. 1982). Every experiment was carried out three times.

### Assays of molecular docking of BSDE compounds

Our models for COX-1 and COX-2 were based on the Protein Data Bank (PDB) structures 3N8X and 5KIR in which the ligands nimesulide and rofecoxib are co-crystallized (Sidhu et al. 2010; Miorando et al. 2024). The APBS-PDB2PQR software (https://server.poissonboltzmann.org/) was used to determine the protonation state of each titrable residue at pH = 7.0 (Jurrus et al. 2018).

The tridimensional structures of constituents from BSDE (taxifolin, quinic acid, chlorogenic acid, α-Boswellic acid, keto-Boswellic acid, and 3-O-acetoxy tirucallic acid) were submitted to the MarvinSketch software to determine the protonation states at pH 7.0. Autodock Vina 1.2 was then used to dock the ligands in the nimesulide and rofecoxib binding sites (Trott and Olson 2010; Eberhardt et al. 2021).

The substrate-nitric oxide synthase complex was investigated using a similar approach, utilizing the 3E7G (Garcin et al. 2018) structure. The heme group, where the iron was pentacoordinated (including by contact with Cys200), and a tetrahydrobiopterin molecule were present in each nitric oxide synthase active site.

### Prediction of physicochemical, pharmacokinetic properties, and toxicological analysis in silico

In this study, we utilized the PubChem (https://pubchem.ncbi.nlm.nih.gov/) and SwissADME (http://www.swissadme.ch) platforms to predict essential physicochemical characteristics of BSDE compounds, focusing on properties related to Lipinski’s Rule of Five. This included evaluating log P (partition coefficient), molecular weight, and the number of hydrogen bond donors and acceptors (Matias Pereira et al. 2021).

The PreADMET (https://preadmet.webservice.bmdrc.org/) website and SwissADME, were used for the prediction of pharmacokinetic (ADME) properties of BSDE. In all analyses, the server’s default parameters were used. Furthermore, a toxicological analysis assessed different toxicity parameters, including mutagenicity, carcinogenicity, and cardiotoxicity (Daina et al. 2017; Nunes et al. 2020).

#### Statistical analysis

All collected data were initially tested using the Shapiro–Wilk normality test to determine normal distributions. All results are expressed as means ± standard error of the means (SEM) for n = 6 animals. Outliers were considered values corresponding to more than 2 standard deviations in relation to the means. Statistical comparisons between groups were performed using one-way analysis of variance (ANOVA) followed by the Bonferroni post hoc test. Analyses were conducted using GraphPad Prism version 5.0 for Windows (GraphPad Software, San Diego, CA, USA). A *p*-value of < 0.05 was considered statistically significant.

## Results

### Chemical analysis

#### Mass spectrometry analysis of BSDE

**Fig. 3:**
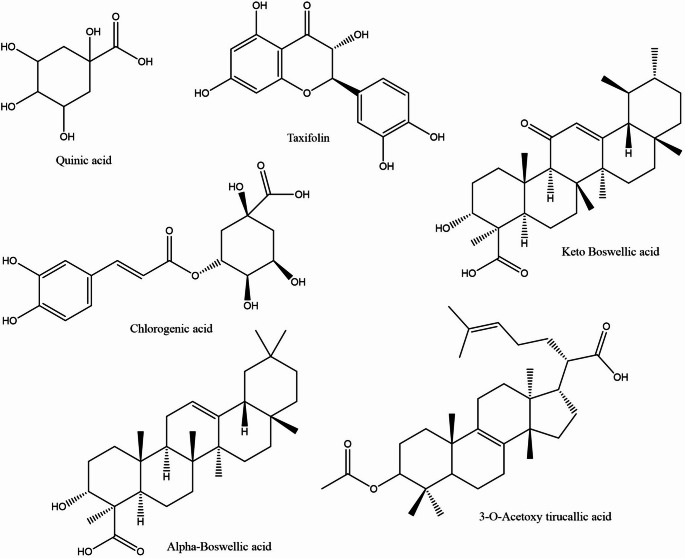
Phytochemical profile of the *Boswellia serrata* dry extract (BSDE) by mass spectrometry analysis (ESI-IT-MS^n^)

**Fig. 4:**
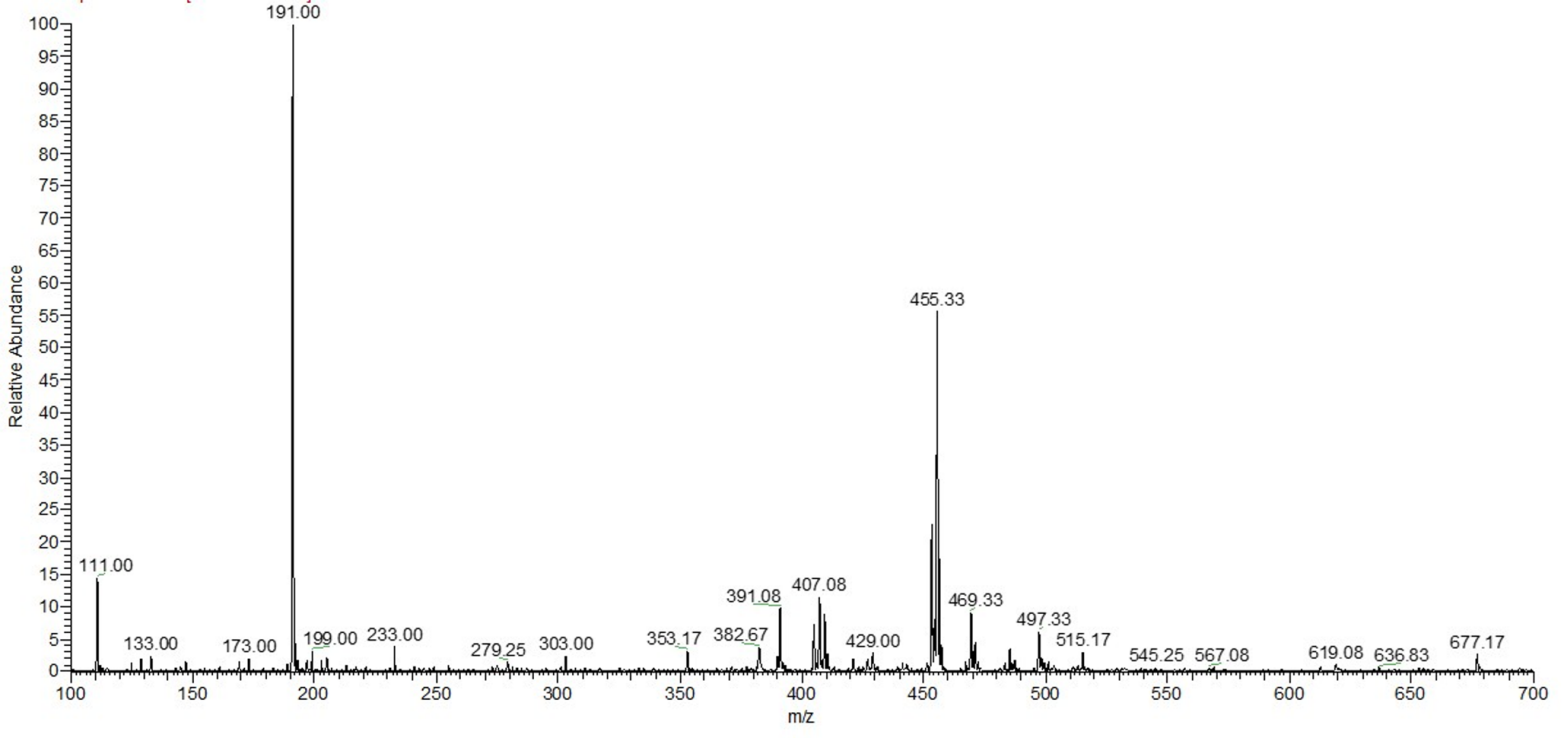
Chemical structures of compounds are present in the *Boswellia serrata* dry extract (BSDE)

As shown in Fig. 3, Six compounds were pointed in the mass spectrometry study of BSDE (ESI-IT-MS^n^). The structures were determined based on their MS2 and MS3 fragmentation patterns and compared with literature data. Among them are α-boswellic acid, keto-boswellic acid, and 3-*O*-acetoxy tirucallic acid (Fig. 4), characteristic of the Boswellia genus (Zhang et al. 2013).

### BSDE accelerates the gastric healing in rats

#### BSDE reduces the macroscopic and microscopic damage in acetic acid-induced ulcers in rats

**Fig. 5:**
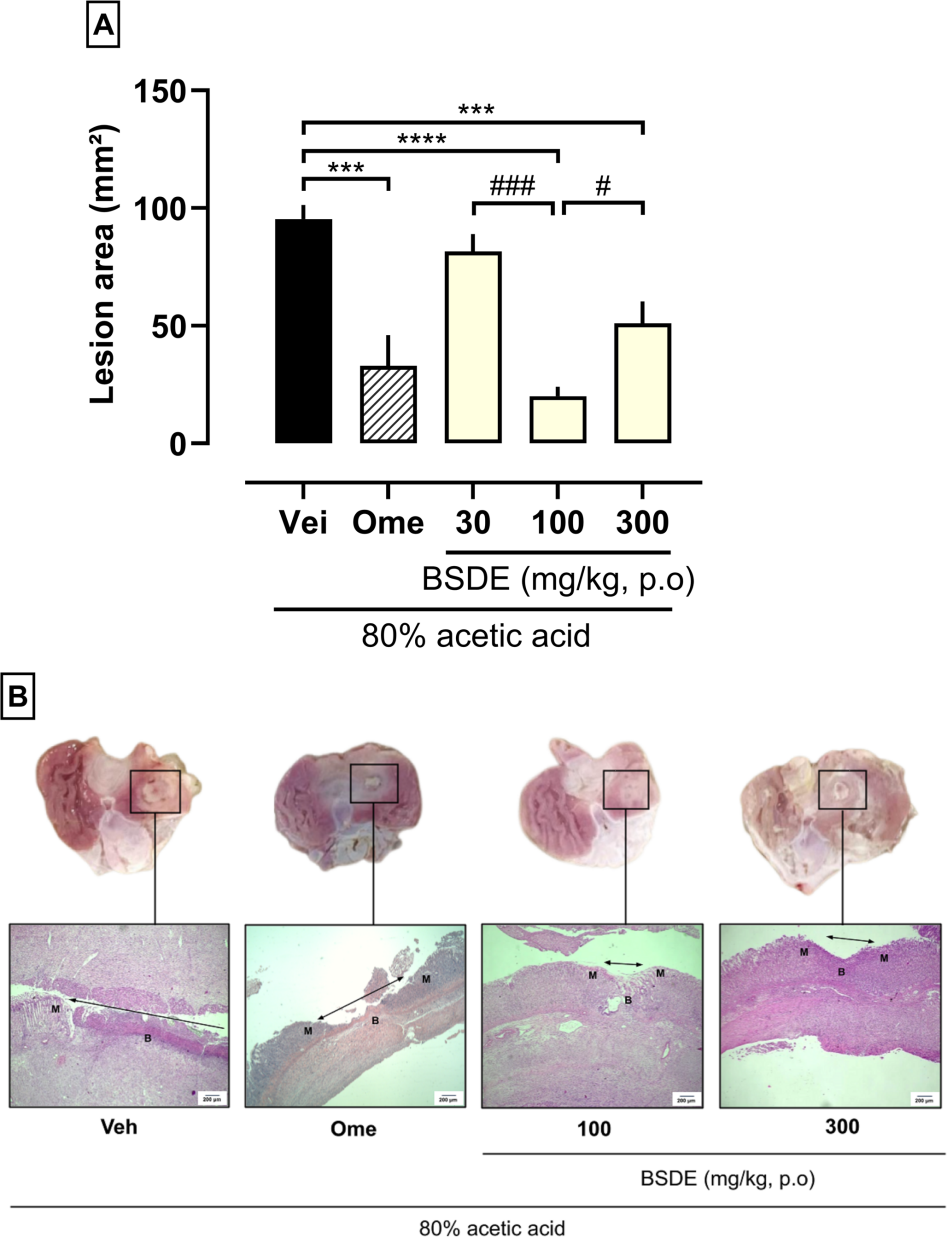
BSDE reduces the ulcer area (**A**) and microscopic damage (**B**) in acetic acid-induced ulcers in rats. The rats were treated with vehicle (water plus 1% tween, 1 mL/kg), omeprazole (20 mg/kg) or BSDE (30, 100 and 300 mg/kg), orally twice daily for 7 days. Panel A: The results were expressed as means ± SEM (n = 6). One-way analysis of variance (ANOVA) followed by Bonferroni’s test was used for statistical analysis. ***P < 0.001 and ***P < 0.0001 compared to the vehicle-treated group and #*P* < 0.05 and ###*P* < 0.001 compared to the group treated with BSDE at 100 mg/kg. Panel B: Representative images of the macroscopic and microscopic lesions in different experimental groups. BSDE: *Boswellia serrata* dry extract

As illustrated in Fig. 5A, rats treated with the vehicle exhibited a mean ulcer area of 95.33 ± 5.98 mm^2^, whereas the administration of BSDE at doses of 100 and 300 mg/kg reduced the lesion area by 79% and 46%, respectively (*p* < 0.0001 and *p* < 0.001 vs. VEH). Omeprazole (OME, 40 mg/kg), used as a reference anti-ulcer drug, reduced the lesion area by 65% compared to the VEH group (*p* < 0.001). Conversely, BSDE at 30 mg/kg did not reduce ulcer size. Representative macroscopic and histological images of these results are shown in Fig. 5B, and it is possible to see that animals treated with BSDE at 100 and 300 mg/kg exhibited attenuated ulcer bases compared to vehicle-treated rats. In parallel, it is also possible to verify a decrease in epithelial damage and inflammatory infiltration in the BSDE-treated groups.

**Fig. 6:**
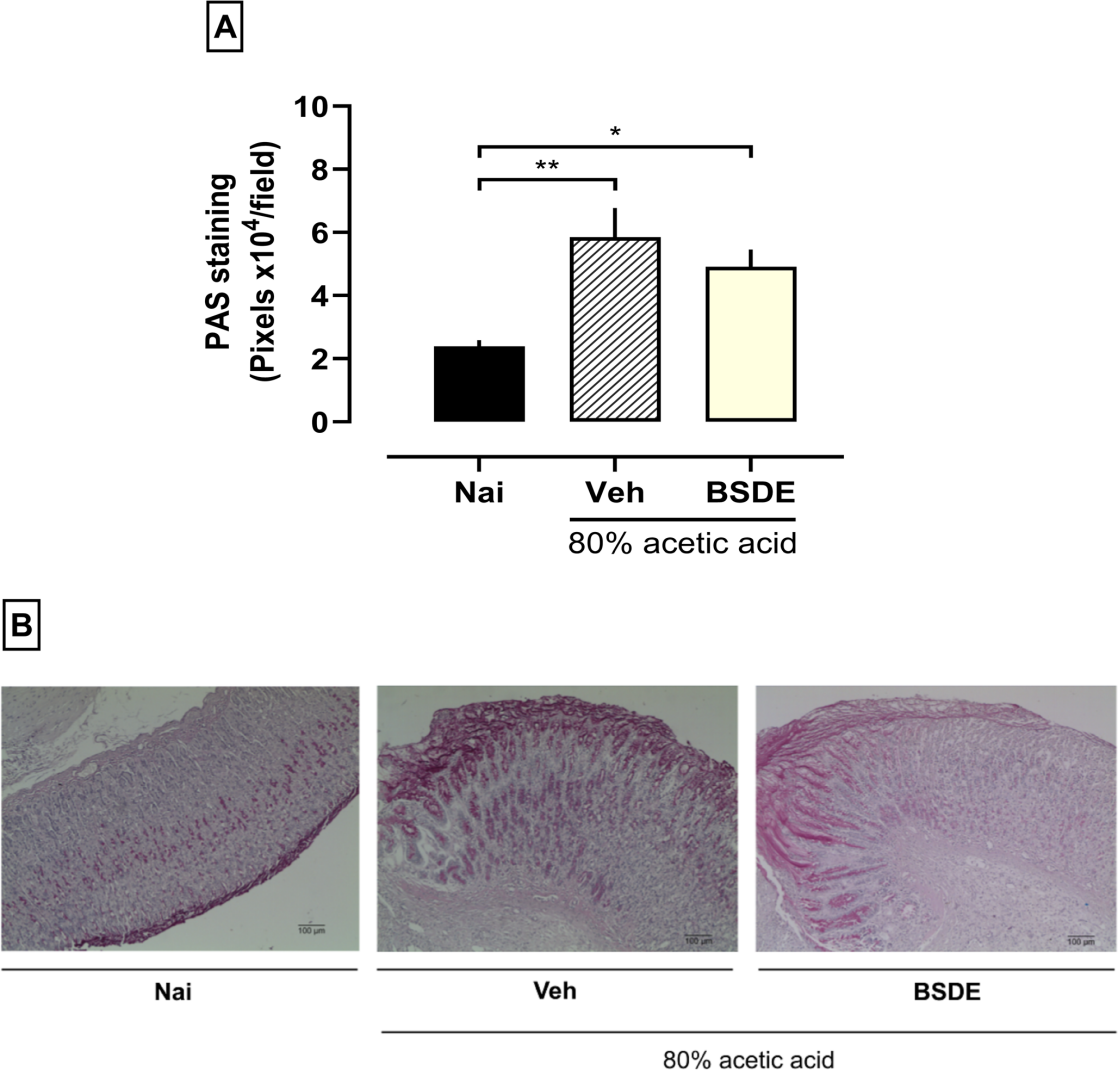
Increased mucin staining by Periodic Acid-Schiff (PAS) in the acetic acid- induced ulcer is observed in vehicle- and BSDE- groups. The rats were treated with water plus 1% tween (1 mL/kg), omeprazole (20 mg/kg) or BSDE (100 mg/kg), orally twice daily for 7 days. Panel A: The results were expressed as means ± SEM (n = 6). One-way analysis of variance (ANOVA) followed by Bonferroni’s post-test was performed. *P < 0.05 and **P < 0.01 compared to the naive group. Panel B: Representative images of the histochemical aspect showing mucins stained in pink by the PAS technique. BSDE: *Boswellia serrata* dry extract

In addition, the mucosal barrier was evaluated by periodic acid-Schiff (PAS) staining to assess the presence of mucins. As shown in Fig. 6A and B, although no differences were found in relation to the vehicle group, BSDE at 100 mg/kg increased the mucin content at the ulcer site twofold when compared to the naive group (2.4 ± 0.2 pixels × 10^4^/field, *p* < 0.05), suggesting greater mucus production.

#### BSDE increases gastric GSH levels and reduces ROS and MDA amount in acetic acid- induced ulcers

**Fig. 7:**
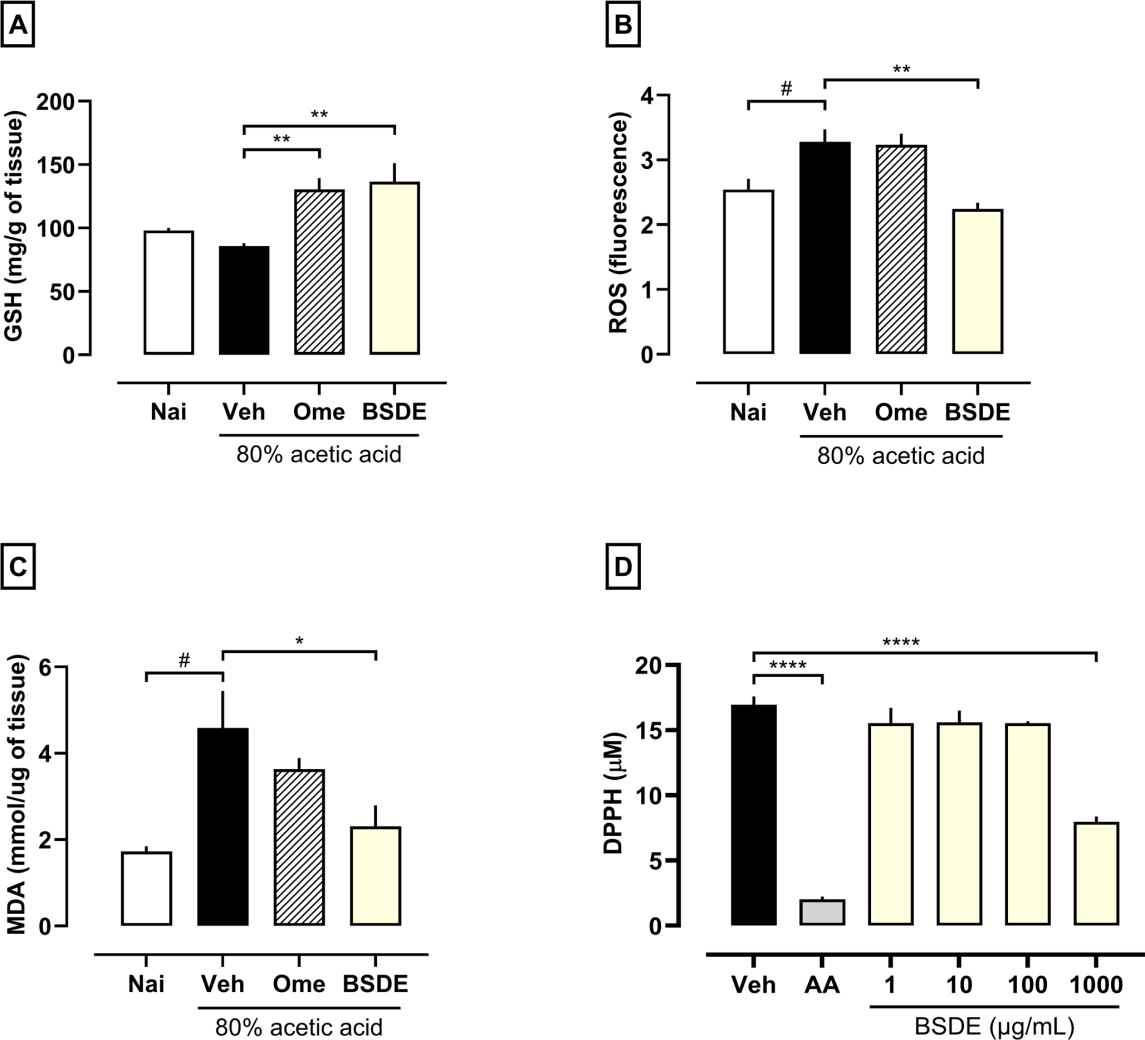
BSDE increases GSH levels (**A**) and reduces ROS (**B**) and MDA (**C**) amount in acetic acid- induced ulcers and shows in vitro antioxidant activity (**D**). Panels A-C: were treated with water plus 1% tween, 1 mL/kg), omeprazole (20 mg/kg) or BSDE (100 mg/kg), orally twice daily for 7 days. Panel D: DPPH radical was incubated with Vehicle (water), Ascorbic acid (AA: 50 μg/mL) or BSDE (1, 10, 100 and 1000 μg/mL). Values are expressed as means ± SEM (n = 6). A one-way analysis of variance (ANOVA) followed by Bonferroni’s test was performed. *P < 0.05, **P < 0.01, and ****P < 0.0001, compared to the respective vehicle-treated group. #*P* < 0.05 compared to the non-ulcerated group (naïve). BSDE: *Boswellia serrata* dry extract

As shown in Fig. 7A, non-ulcerated animals, named naive group, exhibited a mean GSH of 98.09 ± 1.98 µg GSH/g of tissue and ulcerated rats treated with the vehicle showed a mean of 85.80 ± 2.18 µg GSH/g of tissue, and no significant differences were observed between the naive and vehicle-treated groups. In contrast, oral administration of BSDE at dose of 100 mg/kg increased GSH levels by 59% compared to the vehicle group, whereas the treatment with omeprazole (40 mg/kg) increased by 52% the GSH content.

Furthermore, as shown in Fig. 7B, ulcerated animals treated with the vehicle recorded a 29% increase in ROS production compared to non-ulcerated animals (2.5 ± 0.2 fluorescence units, *p* < 0.05). On the other hand, treatment with 100 mg/kg BSDE reduced ROS formation by 32% compared to the vehicle group (3.3 ± 0.2 fluorescence units, *p* < 0.05). In parallel, in Fig. 7C it is possible to observe that MDA amount was increased in ulcerated rats receiving vehicle by 2.65-fold compared to the naive group (1727.0 ± 120.9 mmol/g of tissue, *p* < 0.01). However, rats treated with BSDE at 100 mg/kg showed a reduction of 50% in MDA levels compared to the ulcerated vehicle group, *p* < 0.05.

#### BSDE shows in vitro antioxidant activity only at high concentrations

As shown in Fig. 7D, the incubation of the DPPH radical with BSDE at concentrations ranging from 0.1 to 100 μg/mL exhibited no antioxidant activity in vitro. However, incubation with BSDE at a high concentration (1000 μg/mL) reduced the DPPH radical by 54% compared to the control group (*p* < 0.0001). As expected, the positive control (ascorbic acid at a concentration of 50 μg/mL) decreased the DPPH radical by 88% (*p* < 0.0001).

#### BSDE administration alter SOD, CAT and GST activities in acetic acid- induced ulcers

Table 2 illustrates the effects of ulcer induction and the treatments with BSDE and omeprazole on SOD, CAT, and GST activities. It can be confirmed that the acetic acid ulcer induction increased the SOD and CAT activities in rats treated with vehicle by 28% and 134%, respectively, in comparison to the non-ulcerated group (7.12 ± 0.43 U/mg of protein and 6.03 ± 0.91 mmol H_2_O_2_ consumed/min/mg of tissue, respectively). Regarding the SOD activity the treatment with BSDE at doses of 100 mg/kg reduced this parameter by 24% compared to ulcerated- vehicle group. On the other hand, although BSDE at 100 mg/kg did not change CAT activity. The treatment with omeprazole increased the SOD and CAT activities compared to the vehicle group. GST activity also was measured, and Table 1 shows that no differences were observed between the naive and ulcerated vehicle groups or between the ulcerated group treated with vehicle and BSDE at a dose of 100 mg/kg, but the treatments of ulcerated rats with omeprazole (40 mg/kg) increased the GST activity by 3 times compared to the ulcerated vehicle groups (Table 2).

**Table 1:** Patterns of fragmentation in mass spectrometry (ESI-IT-MS/MS) of the *Boswellia serrata* dry extract (BSDE)

Compound	*m/z* [M-H] ^–^	MS/MS	References
Quinic acid	191	173, 111, 85	Zanatta et al. (2021)
Taxifolin	303	285, 275, 241, 199, 125	Chen et al. (2016)
Chlorogenic acid	353	173, 111	Willems et al. (2016)
α-Boswellic acid	455	437, 409, 361, 233	Katragunta et al. (2019)
Keto boswellic acid	469	407, 391	Katragunta et al. (2019)
3-*O*-Acetoxy tirucallic acid	497	437, 415, 397, 355	Katragunta et al. (2019)

**Table 2:** Effects of BSDE in superoxide dismutase (SOD), catalase (CAT) and Glutathione S-transferase (GST) activities at gastric ulcer site

	SOD	CAT	GST
	(U/mg of protein)	(mmol H_2_O_2_/min/mg of protein)	(mmol GSH/min/mg of protein)
Naive	6.0 ± 0.9	7.1 ± 0.4	0.32 ± 0.06
Vehicle	14.1 ± 2.2^a^	9.1 ± 0.8^a^	0.24 ± 0.06
Omeprazole	5.9 ± 1.1	9.7 ± 0.6^d^	0.73 ± 0.09^b,e^
BSDE	10.7 ± 0.9^c^	6.9 ± 0.4	0.46 ± 0.09

The animals received orally vehicle (water plus 1% tween, 1 mL/kg), Omeprazole (20 mg/kg) or BSDE (100 mg/kg). Values are expressed as mean ± SEM (n = 6). The results were subjected to statistical comparison using one-way analysis of variance (ANOVA) followed by Bonferroni’s post-hoc test. Significant differences were denoted as ^a^*P* < 0.05 and ^b^*P* < 0.01 compared to the naive group. ^c^*P* < 0.05, ^d^*P* < 0.01 and ^e^*P* < 0.001 compared to the ulcerated group treated with the vehicle

#### BSDE administration reduces MPO activity in acetic acid- induced ulcers

**Fig. 8:**
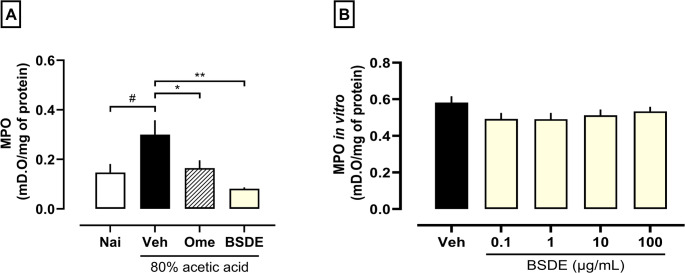
BSDE reduces in vivo (**A**), but not in vitro (**B**), MPO activity in acetic acid- induced ulcers. Values are expressed as means ± SEM (n = 6). A one-way analysis of variance (ANOVA) followed by Bonferroni’s test was performed. *P < 0.05, and **P < 0.01 compared to the vehicle-treated group. #*P* < 0.05 compared to the non-ulcerated group (naïve). BSDE: *Boswellia serrata* dry extract

Figure 8A displays the results of MPO activity, an inflammatory marker. The average MPO activity of non-ulcerated animals was 0.15 ± 0.03 mD.O./mg of protein, while the ulcerated rats treated with a vehicle had a 100% increase in MPO activity when compared to the non-ulcerated group. On the other hand, the MPO activity was decreased to levels equivalent to those observed in the non-ulcerated group by treatment with omeprazole (40 mg/kg) or BDSE at 100 mg/kg. About the ability of BSDE to act directly on MPO activity, no differences were observed in the in vitro incubation of different concentrations of BSDE compared to the vehicle (0.6 ± 0.03 mD.O./mg of protein, Fig. 8B).

### BSDE reduces the colitis severity in rats

#### BSDE decreases the shortening of colon in acetic acid-induced colitis in rats

**Fig. 9:**
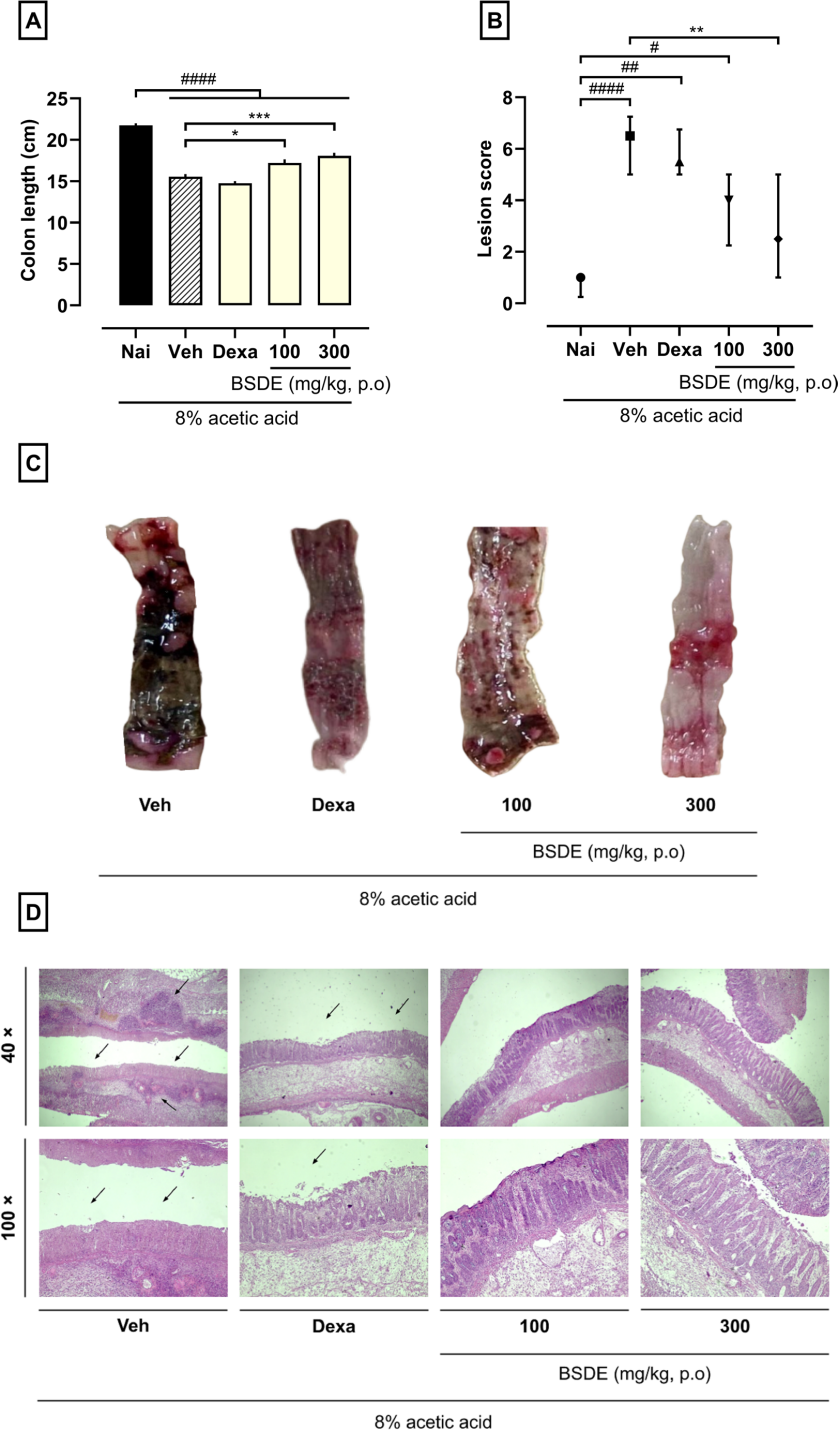
BSDE preserves colon length (**A**) and reduces macroscopic (**B** and **C**) and microscopic (**D**) lesion score of acetic acid- induced colitis in rats. Panel A: Values were expressed as means ± SEM (n = 6). One way analysis of variance (ANOVA) followed by Bonferroni’s multiple comparison test. ####*P* < 0.0001, compared with the non-colitic group (naïve) and *P < 0.05, and ***P < 0.001 compared to the colitic vehicle-treated group (Veh: (water plus 1% tween, 1 mL/kg, p.o). Panel C: Representative images from the colonic appearance of rats from experimental groups. Dexa: dexamethasone (2 mg/kg, s.c.). BSDE: *Boswellia serrata* dry extract

As shown in Fig. 9A, the average colon length in the naïve group (non-colitic rats) was 21.75 ± 0.25 cm. In contrast, animals treated with vehicle or dexamethasone exhibited significant colon shortening, with reductions of 28.50% and 32.18%, respectively, compared to the naïve group. Notably, treatment with BSDE at 100 and 300 mg/kg resulted in an increase in colon length of 11% and 16%, respectively, when compared to the vehicle-treated group.

#### BSDE administration reduces the macroscopic and microscopic lesion score of acetic acid- induced colitis in rats

Figure 9B shows that as compared to the vehicle group, BSDE decreased colonic damage scores. In comparison to the vehicle group, the injury score was reduced 1.68 and 1.92 times, respectively, by treatment with 100 and 300 mg/kg BSDE. Remarkably, in comparison to the vehicle, dexamethasone (2 mg/kg) had no effect on injury scores. It is possible to confirm that the colons of the animals receiving vehicle treatment showed widespread ulceration, hyperemia, hemorrhagic lesions, and inflammation by looking at the typical macroscopic images of the last 6 cm of the proximal colon for each experimental group shown in Fig. 9C. On the other hand, these lesions were visibly attenuated in groups treated with BSDE, especially at the higher dose.

Furthermore, several histopathological changes were noted in animals treated to the vehicle, including loss of mucosal and crypt architecture, submucosal edema, leukocyte infiltration, and superficial ulceration, as shown in Fig. 9D, which shows sections of the colon stained with hematoxylin and eosin. Animals treated with dexamethasone showed a similar pattern. Nevertheless, groups treated with BSDE, especially at 300 mg/kg, showed histological improvements, including decreased edema, reduced leukocyte infiltration, and retained epithelial integrity.

#### BSDE increases mucin PAS- staining in acetic acid-induced colitis in rats

**Fig. 10:**
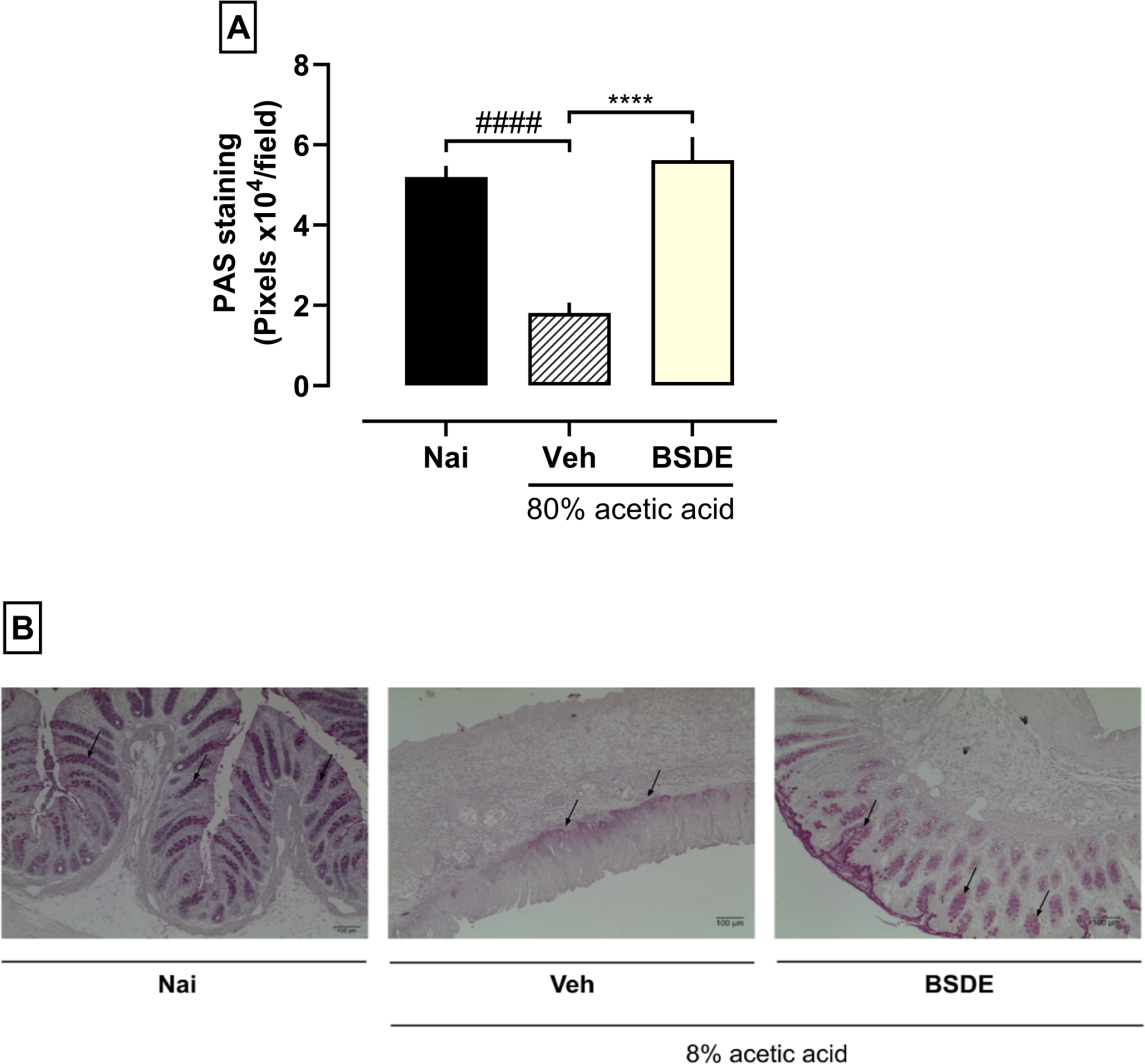
BSDE increases mucin staining by Periodic Acid-Schiff (PAS) in the acetic acid- induced colitis. The rats were treated 3 days before and 3 days after colitis induction with water plus 1% tween (1 mL/kg, p.o), Dexamethasone (Dexa, 2 mg/kg, s.c) or BSDE (100 mg/kg, p.o). Panel A: The results were expressed as means ± SEM (n = 6). One-way analysis of variance (ANOVA) followed by Bonferroni’s post-test was performed. ****P < 0.0001 and ####*P* < 0.0001 compared to the vehicle and naive group, respectively. Panel B: Representative images of the histochemical aspect showing mucins stained in pink by the PAS technique. BSDE: *Boswellia serrata* dry extract

Regarding the mucus barrier, as shown in Fig. 10A and B, instilling 8% acetic acid into the colonic mucosa of animals treated with the vehicle reduced mucin production by 65%, compared to the non-colitic group (5.2 ± 0.3 pixels × 10^4^/field, *p* < 0.0001). However, treatment with 300 mg/kg of BSDE increased mucin production threefold compared to the vehicle-treated colitic group (1.8 ± 0.3 pixels × 10^4^/field, *p* < 0.0001).

#### BSDE reduces the oxidative stress in of acetic acid- induced colitis in rats

**Fig. 11:**
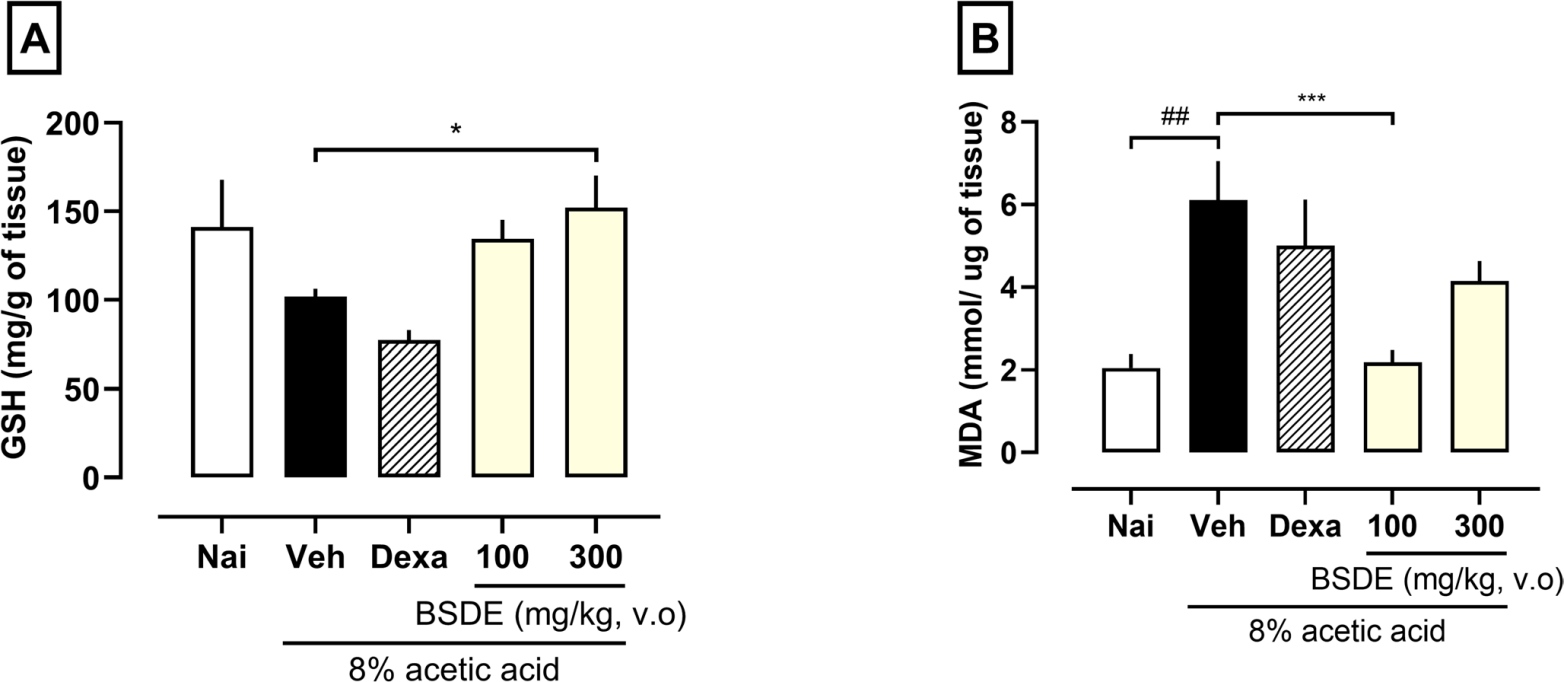
BSDE reduces the oxidative stress in of acetic acid- induced colitis in rats. The rats were treated 3 days before and 3 days after colitis induction with water plus 1% tween (1 mL/kg, p.o), Dexamethasone (Dexa, 2 mg/kg, s.c) or BSDE (100 mg/kg, p.o). Panel A: The results were expressed as means ± SEM (n = 6). One-way analysis of variance (ANOVA) followed by Bonferroni’s post-test was performed. ****P < 0.0001 and ####*P* < 0.0001 compared to the vehicle and naive group, respectively. Panel B: Representative images of the histochemical aspect showing mucins stained in pink by the PAS technique. BSDE: *Boswellia serrata* dry extract

GSH levels in animals treated with vehicles and dexamethasone were reduced by 27.88 and 45.56% in comparison to the naïve group (141.3 ± 19.78 μg GSH/g tissue), as shown in Fig. 11A. On the other hand, GSH levels increased by 45% after treatment with BSDE at 300 mg/kg in comparison to the vehicle group (105.00 ± 5.05 μg GSH/g tissue). However, in comparison to the vehicle or naïve groups, BSDE at 100 mg/kg did not significantly change GSH levels.

The MDA levels in the vehicle group were 3.14 times higher than those in the naïve group (2.04 ± 0.25 mmol/mg tissue), as seen in Fig. 11B as well. MDA levels in rats treated with dexamethasone were likewise 2.44 times higher than in the naïve group. Additionally, MDA levels were reduced by 55% and 41%, respectively, by BSDE at 100 and 300 mg/kg in comparison to the vehicle group (6.44 ± 0.99 mmol/mg tissue).

#### Molecular docking analyses of BSDE

**Fig. 12:**
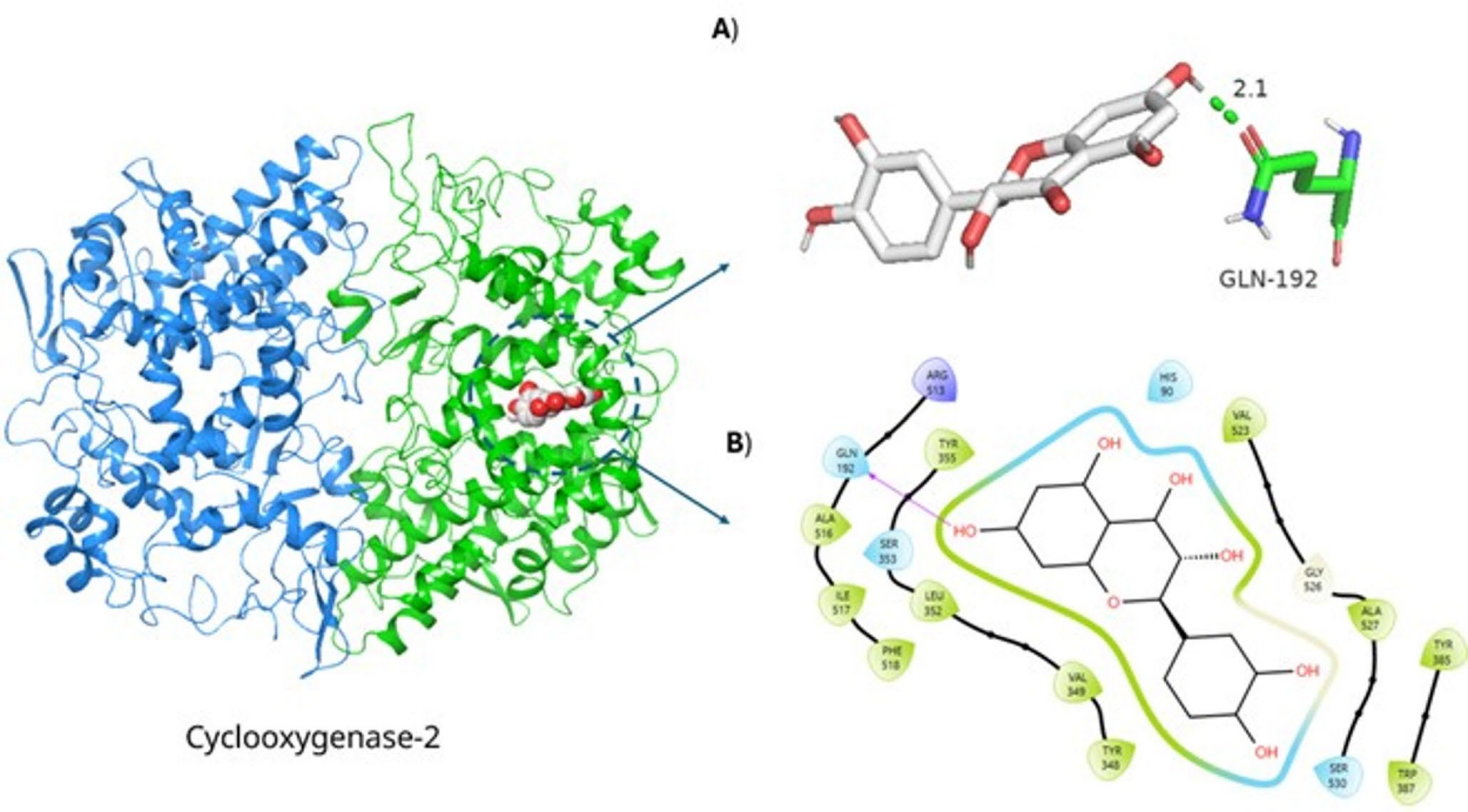
Best docking pose and interaction profiles of taxifolin present in Boswellia serrata dry extract (BSDE) with Cyclooxygenase-2 (PDB: 5KIR). **A** Taxifolin 3D interacting with residue Gln192 through a hydrogen bond; **B** Taxifolin 2D *interacting* with other amino acid residues

Table 3 presents the docking binding energies of the ligands to Cyclooxygenase-1 (COX-1). The protonation state of the ligands and the protein’s titrable residues match at pH = 7.0. Although the docking energies are remarkably similar, the protein has a greater affinity for our compounds (especially chlorogenic acid) than for the co-crystallized ligand, nimesulide. Unlike rofecoxib, which formed hydrogen bonds with Ile517 and Phe518, taxifolin revealed the best binding energy (− 9.3 kcal/mol) with COX-2 through the Gln192 residue. This interaction of the bioactive compound with the active site of the enzyme occurred essentially through the hydroxyl of the catechol group (Fig. 12).

**Table 3:** Molecular affinity parameters of the compounds *Boswellia serrata* dry extract (BSDE) with the enzymes Cyclooxygenase-1(PDB: 3N8X), Cyclooxygenase-2 (PDB: 5KIR), and inducible enzyme nitric oxide synthase (PDB: 3E7G)

Compounds	3N8X- ∆G (kcal/mol)	5KIR—∆G (kcal/mol)	3E7G—∆G (kcal/mol)
Quinic acid	− 6.5	− 6.4	− 6.1
Taxifolin	− 8.3	− 9.3	− 9.5
Chlorogenic acid	− 8.8	− 8.9	− 9.2
α-Boswellic acid	− 8.4	− 8.5	− 9.5
Keto boswellic acid	− 8.4	− 8.3	− 9.4
3-*O*-Acetoxy tirucallic acid	− 8.0	− 7.8	− 9.5
Nimesulide	− 8.2	–	–
Rofecoxib	–	− 10.2	–
AR-C95791	–	–	− 8.8

Nimesulide, rofecoxib, and AR-C95791, Ethyl 4-[(4-Methylpyridin-2-YL)Amino]Piperidine-1-Carboxylate (co-crystallizeds ligands)

**Fig. 13:**
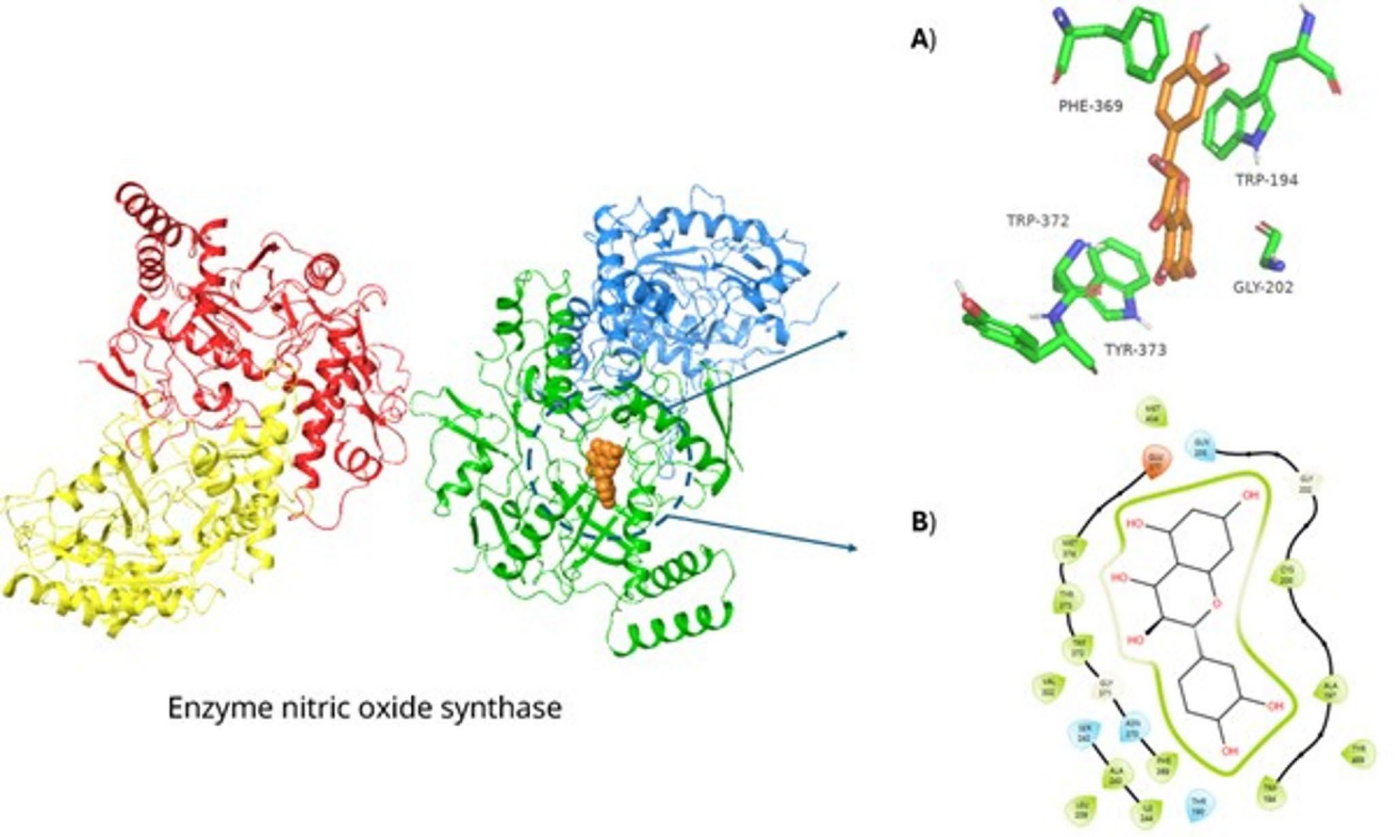
Best docking pose and interaction profiles of taxifolin present in Boswellia serrata dry extract (BSDE) with nitric oxide synthase (PDB: 3E7G). **A** Structure of the most stable ligand (taxifolin)-nitric oxide synthase complex as predicted by molecular docking; **B** The ligand and residues that interact via hydrophobic bonds are shown in intensely green lines

The interaction of BSDE with the inducible nitric oxide synthase enzyme demonstrated comparable binding energies, with taxifolin and 3-*O*-Acetoxy tirucallic acid exhibiting the highest binding affinities (− 9.5 kcal/mol, respectively) (Table 2). The interactions between the phytoconstituents and the enzyme involve the residues Trp194, Gly202, Phe369, and Tyr373 (Fig. 13). On the other hand, the co-crystallized ligand ethyl 4-[(4-methylpyridin-2-yl) amino] piperidine-1-carboxylate (AR-C95791) showed lower binding energy (− 8.8 kcal/mol) with the enzyme.

#### Prediction of physicochemical, pharmacokinetic properties, and toxicological analysis

The compounds of BSDE exhibited favorable characteristics of bioavailability (Table 4), with no violations of Lipinski’s rule or only one violation. These results suggest that the molecules have a promising profile for potential drug candidates. Furthermore, it is essential to note that successful agents derived from natural products often display two to three violations of these rules (Benet et al. 2016), suggesting that the presence of some exceptions may not impede the therapeutic efficacy of these compounds (Bultum et al. 2022).

**Table 4:** Physicochemical properties for the compounds present in BSDE

Compound	Molecular weight (Dalton)	Hydrogen bond donor	Hydrogen bond aceptor	Log P^a^	RO5 Violations^b^
Quinic acid	192.17	5	6	− 2.4	0
Taxifolin	304.25	5	7	1.5	0
Chlorogenic acid	354.31	6	8	− 0.4	1
α-Boswellic acid	456.71	2	3	9.43	1
Keto boswellic acid	470.7	2	4	7.2	1
3-*O*-Acetoxy tirucallic acid	449.35	1	4	4.45	0

^a^Lipophilicity of compounds expressed by partition coefficient, calculated by PubChem and SwissADME

^b^Number of violations of Lipinski’s Rule of Five. CID, PubChem Compound Identification

In silico ADMET predictions, while not completely replacing traditional in vitro and in vivo approaches, offer significant insights into toxicity and ADME (Absorption, Distribution, Metabolism, and Excretion) properties (Moroy et al. 2012; Espinoza-Culupú et al. 2023). The results from Table 5 indicate that the compounds of BSDE, except for quinic acid, present a moderate to high intestinal absorption rate (HIA). Furthermore, plasma protein binding (PPB) analysis demonstrated a high percentage of binding for taxifolin, 3-*O*-acetoxy tirucallol, α-boswellic acid, and keto-boswellic acid. At the same time, the ability to traverse the intestinal barrier was confirmed for *α*-Boswellic acid, Keto boswellic acid, and 3-O-Acetoxy tirucallic acid in permeability measurements in Caco-2 and MDCK cells.

**Table 5:** Prediction of pharmacokinetic properties of molecules present in BSDE, using the PreADMET and SwissADME website

Compounds	HIA^a^ (%)	Plasma Protein Binding	pCaco-2^b^ (nm/sec)	pMDCK^c^ (nm/sec)	BBB^d^
Quinic acid	20.2869	6.5950	8.2724	0.5302	0.4285
Taxifolin	60.1637	95.1644	3.4230	9.5674	0.1669
Chlorogenic acid	20.4278	41.9617	18.7168	4.5134	0.0336
α-Boswellic acid	95.9962	100	21.8690	0.0441	7.8709
Keto boswellic acid	96.7140	100	21.3932	0.0438	2.4577
3-*O*-Acetoxy tirucallic acid	97.8254	98.1881	24.9461	0.04387	3.7541

^a^Human intestinal absorption

^b^Cell permeability in Caco-2

^c^Cell permeability MDCK

^d^Penetration of the blood–brain barrier

The toxicity of the compounds depends on the dose and duration of exposure, which may manifest differently among cell lines and physiological states (Knudsen et al. 2020). Using computational techniques enables efficient drug screening to be completed in less time and at a lower cost. In this study, toxicity assessments were classified through tests such as the Ames test, carcinogenicity in rodents, and hERG channel inhibition, revealing that *α*-Boswellic acid, 3-*O*-Acetoxy tirucallic acid, and Keto boswellic acid present a low risk of toxicity (Table 6).

**Table 6:** Toxicity prediction for compounds of BSDE using the PreADMET website

Compound	Ames_test	Carcino_mouse	Carcino_rat	hERG_inhibition
Quinic acid	Mutagem	Alerts^a^	No alerts^b^	Low risk
Taxifolin	Mutagem	Alerts	No alerts	Medium risk
Chlorogenic acid	Mutagem	No alerts	Alerts	Medium risk
α-Boswellic acid	Non-mutagem	No alerts	No alerts	Low risk
Keto boswellic acid	Non-mutagem	No alerts	No alerts	Low risk
3-*O*-Acetoxy tirucallic acid	Non-mutagem	NO alerts	No alerts	Low risk

^a^Clear evidence of carcinogenic activity

^b^No evidence of carcinogenic activity

## Discussion

Ayurvedic and traditional Chinese medicine have long used *B. serrata*, a tree indigenous to dry parts of India, Africa, and the Middle East, to relieve pain and inflammation. Because its oleo-gum resin contains bioactive pentacyclic triterpenes, particularly boswellic acids, it has therapeutic benefits. Given their immunomodulatory, antioxidant, and anti-inflammatory properties, these substances have shown great pharmaceutical promise (Chauhan et al. 2021). In two experimental models that closely resemble human gastrointestinal disorders the current study confirmed the dual experimental efficacy of a dry extract of *B. serrata* (BSDE), standardized to 65% boswellic acids, mitigating the intestinal inflammation and accelerating the gastric healing damage.

The study’s findings collaborate with the validation of *B. serrata*'s traditional ethnomedical applications in gastrointestinal disorders and further supports its potential as a treatment for inflammatory and ulcerative digestive tract disorders. Moreover, since most anti-inflammatory medications on offer today have detrimental effects on the gastric mucosa rather than beneficial ones, the evidence presented here that rats treated with BSDE exhibit improvements in intestinal anti-inflammation and accelerated stomach repair provides an interesting insight into the traditional use of this plant as an anti-inflammatory remedy with gastric tolerability.

With the benefits of an easy induction procedure, excellent repeatability, and a high survival rate, the acetic acid-induced ulcer model is one of the most widely used models and is well-known in the scientific community due to its pathological morphology and repair process being like that of human gastric ulcers (Okabe and Amagase 2005). As a result, the findings from this research showed that *B. serrata* had a therapeutic effect on gastric ulcers, with improved recovery outcomes noted for a variety of parameters.

The ulcer area was decreased by BSDE treatment at 100 and 300 mg/kg; however, it is noteworthy that the reduction reached at 100 mg/kg was significantly more effective than those seen in rats given 300 mg/kg of the extract (*p* < 0.05). Therefore, to continue the research, we chose a dose of 100 mg/kg to investigate the extract’s mode of action. This data indicates that in the context of gastric damage the extract can act following a hormetic dose–response curve, where low doses display better effects. Indeed, Lewinska et al. (2019) demonstrated that a natural derived from boswellic acid showed biphasic hormetic dose response by stimulating cell growth, survival and metabolic activity in fibroblasts at low doses (up to 1 μM), while showing inhibitory effects at high doses (> 10 μM). Indeed, the presence of boswellic acid in BSDE was confirmed during the phytochemical analysis and fibroblasts are crucial to the ulcer repair, therefore the findings from Lewinska et al. (2019) can contribute to the explaining the better results achieved at 100 mg/kg.

Accompanying the macroscopic findings, the histological examination revealed better mucosal integrity and smaller ulcer bases in the BSDE group treated with 100 mg/kg. Periodic acid–Schiff (PAS) staining further indicated an increase in mucin production in the 100 mg/kg group, suggesting that BSDE may facilitate ulcer healing by enhancing mucosal protective factors.

The healing effects observed were closely associated with the extract’s ability to modulate oxidative stress, a known contributor to the pathogenesis of peptic ulcers. Notably, BSDE increased GSH levels, which is critical for preserving cellular redox homeostasis. Concurrently, it decreased MDA levels, a marker of lipid peroxidation, indicating reduced oxidative membrane damage. Additionally, BSDE normalized SOD activity, which was elevated in the vehicle group, and maintained catalase CAT activity closer to the found in non-ulcerated group. These findings align with prior reports on the antioxidant properties of *B. serrata* resin and support its potential to mitigate oxidative mucosal injury (Hartmann et al. 2012, 2014). The ability of BSDE to scavenge a free radical was tested based on the in vivo antioxidant system results; however, no decreases in DPPH levels were seen because of the extract’s incubation. This suggests that the improvement in the oxidative status of ulcerated gastric mucosa that BSDE promotes is not directly caused by scavenging free radicals, but rather by the enhancement of endogenous antioxidant systems or the decrease in endogenous reactive species production.

Importantly, MPO activity, a marker of neutrophil infiltration and inflammation, was significantly reduced in BSDE-treated group, indicating attenuation of the inflammatory response at the gastric level. Activated leukocytes have been implicated in gastric mucosal injuries induced by different agents (Harada et al. 1999; Valois et al. 2021) and it is known that activated leukocytes release a variety of inflammatory mediators, including granulocyte elastase and ROS that damage the endothelial cells and other tissues (Harada et al. 1999). According to the MPO activity results, the improvement in oxidative stress may therefore be the result of a decrease in ROS production that is mediated by active neutrophils. Furthermore, the in vitro incubation of a sample with higher levels of MPO activity with the extract failed to produce any changes, indicating that the in vivo results in the decrease of MPO are due to a reduction of neutrophil migration to the gastric tissue. As a result, the direct inhibition of MPO mediated by BSDE was discarded.

In the colitis model, BSDE similarly demonstrated potent anti-inflammatory effects, corroborating the findings from Hatmann et al. (2012, 2014). Macroscopically, BSDE treatment reduced tissue damage induced by acetic acid, and these improvements were paralleled by reductions in MPO activity in colonic tissue. Again, the ability of BSDE to decrease MPO suggests effective modulation of neutrophil recruitment and activity at the inflamed mucosal site, which can repercuss in reduced oxidative damage, evidenced by the decrease in MDA levels together with the increase in GSH availability at colon from rats treated with BSDE.

The pentacyclic triterpenic acids isolated from various *Boswellia* species are collectively called Boswellic Acids (BA). Previous investigations into pharmacological studies have emphasized the efficacy of BA in managing various chronic inflammatory diseases, including chronic ulcerative colitis, rheumatoid arthritis, Crohn’s disease, and bronchial asthma (Zhang et al. 2013). In line with these findings, we highlight α-boswellic acid, keto-boswellic acid, and 3-*O*-acetoxy-tirucallic acid in BSDE. In addition, we detected quinic, chlorogenic acids, and taxifolin, phenolic compounds recognized for their immunomodulatory, anti-inflammatory, and antioxidant activities (Sun and Shahrajabian 2023).

BA exerts their anti-inflammatory effects through multiple mechanisms, distinguishing them from conventional NSAIDs. Rather than inhibiting cyclooxygenase (COX), they inhibit the 5-lipoxygenase (5-LO) pathway, leading to a reduction in leukotriene synthesis, a critical mediator in chronic inflammation and autoimmune disease (Poeckel and Werz 2006). Moreover, BA suppress the activation of nuclear factor-kappa B (NF-κB), a transcription factor involved in cytokine production (TNF-α, IL-1β, IFN-γ) and perpetuation of inflammatory cascades (Ding et al. 2014). This multi-targeted mechanism likely explains the extract’s efficacy in the present study in controlling both gastric and intestinal inflammation.

According to De Souza Neto et al. (2020), in silico research facilitates the prediction of characteristics and factors (including pharmacokinetics, toxicity, and pharmacodynamics) that are suitable for the development of drug candidates. Moreover, according to Shaker et al. (2021), this investigation demonstrated that BSDE has advantageous ADME characteristics and a favorable toxicity profile.

Additionally, we employed docking simulations to elucidate the mechanism by which BSDE maintains mucin in epithelial cells and regulates nitric oxide homeostasis. The compounds interacted efficiently with the active sites of the COX-1 and COX-2 enzymes, and their protective effects on the mucosa were observed in vivo, with the maintenance of mucus and demonstration of anti-inflammatory effects.

In our analysis, we observed that the special taxifolin formed hydrogen bonds with COX-2 in the residue of Gln192 and got very close to Ser530 (OH in the para position of the catechol ring), where the classical AINES perform the interactions (Finamore et al. 2019). The data together can contribute to preventing the synthesis of prostaglandins via inducible pathways. This finding provides evidence for understanding the protection of the stomach mucosa exerted by BESD.

Additionally, NO contributes to the integrity of the mucosa by providing adequate blood flow and mucus secretion (Wallace and Miller 2000). However, NO produced by the action of the inducible nitric oxide synthase (iNOS) is related to gastric injury induced by NSAIDs and ethanol (Lee et al. 2005). In this investigation, taxifolin was found to have essential interactions with the binding site of iNOS, with can mitigates enzymatic activity and partially explaining the role of NO in the gastric protective activity of BESD.

From an ethnopharmacological perspective, the historical use of *B. serrata* for gastrointestinal complaints in traditional medicine gains further credibility through these findings. The evidence supports the hypothesis that the standardized extract (BSDE) could serve as a viable phytotherapeutic option, offering both gastric mucosal protection and modulation of intestinal inflammation. Its dual action—enhancing endogenous antioxidant defenses while suppressing inflammatory mediators—provides a rational basis for its integration into modern complementary treatments for peptic ulcer disease and IBD.

However, this study presents certain limitations, primarily due to its preclinical design, which may not fully reflect the complexity of human pathophysiology. Moreover, the lack of standardization of herbal extracts and the compositional variability among commercially available *Boswellia* supplements represent significant barriers to their widespread clinical application. Consequently, randomized, controlled clinical trials in human populations are essential to validate the efficacy and safety of *B. serrata* dry extract (BSDE).

## Conclusion

In conclusion, the dry extract of *Boswellia serrata* (BSDE) exhibited anti-ulcer and anti-colitic activities in nonclinical models, supporting its traditional use in gastrointestinal disorders. The observed therapeutic effects, likely mediated through anti-inflammatory, antioxidant, and mucosal-protective mechanisms, underscore its potential as a safe and multi-targeted intervention for gastrointestinal inflammation. These findings contribute to the expanding body of evidence advocating for the rational integration of medicinal plant-based therapies into evidence-based clinical practice.

## Data Availability

Data will be made available on request.
