# Correction: *Boswellia serrata* dry extract with intestinal anti-inflammatory properties also accelerates gastric ulcer healing in rats

**DOI:** 10.1007/s10787-026-02263-w

**Published:** 2026-06-22

**Authors:** Franco Giovanni Sandri Serafim, Lucas Fontana Breguez da Cunha, Larissa Venzon, Ana Carolina dos Santos Nilz, Bruna Longo, Ruan Kaio Silva Nunes, Max Vidal Gutiérrez, Daniela Miorando, Cristian A. Dalla Vecchia, Walter Antônio Roman Junior, Luisa Mota da Silva

**Affiliations:** 1 School of Health, University of Vale do Itajaí, Itajaí, SC Brazil; 2 Postgraduate Program in Pharmaceutical Sciences, University of Vale do Itajaí, Itajaí, SC Brazil; 3 TGI Pharmacology and Its Interactions Laboratory, Department of Pharmacology, Federal University of Santa Catarina (UFSC), R. João Pio Duarte Silva, 241 - Córrego Grande, Florianópolis, SC 88037-000 Brazil; 4 Department of Chemical, Biological and Agricultural Sciences, University of Sonora, Navojoa Sonora, Mexico; 5 Postgraduate Program in Health Sciences, Community University of Chapecó Region, Chapecó, SC CEP 89809-900 Brazil

**Correction to: Inflammopharmacology**

 10.1007/s10787-026-02133-5

In this article, the Fig. 5 has been incorrectly published in the original version. The complete correct Fig. 5 is given below.

**Fig. 5:**
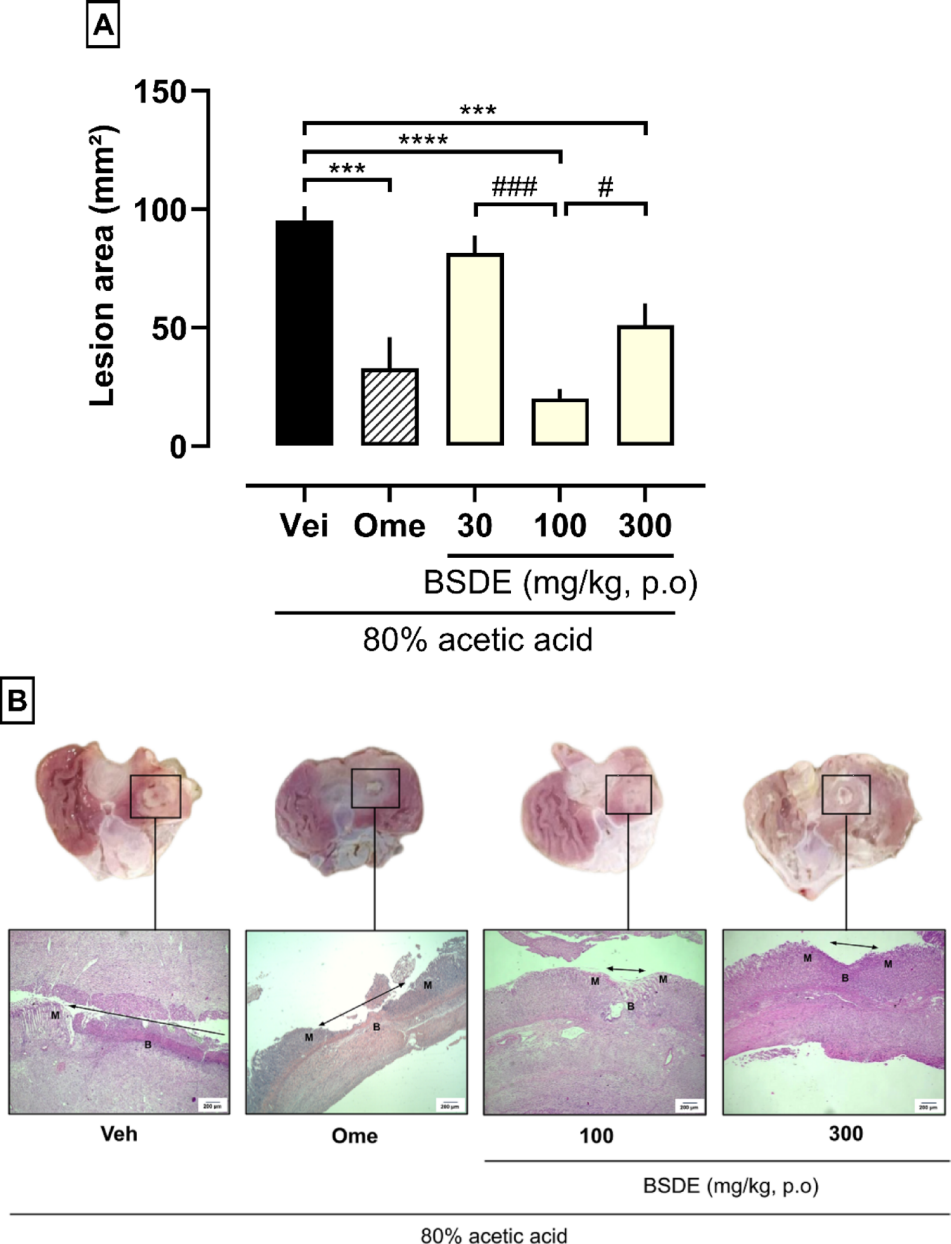
BSDE reduces the ulcer area (**A**) and microscopic damage (**B**) in acetic acid-induced ulcers in rats. The rats were treated with vehicle (water plus 1% tween, 1 mL/kg), omeprazole (20 mg/kg) or BSDE (30, 100 and 300 mg/kg), orally twice daily for 7 days. Panel A: The results were expressed as means ± SEM (n = 6). One-way analysis of variance (ANOVA) followed by Bonferroni’s test was used for statistical analysis. ***P < 0.001 and ***P < 0.0001 compared to the vehicle-treated group and #*P* < 0.05 and ###*P* < 0.001 compared to the group treated with BSDE at 100 mg/kg. Panel B: Representative images of the macroscopic and microscopic lesions in different experimental groups. BSDE: *Boswellia serrata* dry extract

The original article has been corrected.

